# Lost in translation: a qualitative study of medical students’ experiences of theoretical and practical teaching of empathy

**DOI:** 10.1186/s12909-024-06385-z

**Published:** 2024-12-04

**Authors:** Johanna von Knorring, Johanna Salmi, Arja Lehti, Olof Semb

**Affiliations:** 1 Department of Clinical Sciences, Professional Development, Umeå University, Umeå, Sweden; 2 Present Address: Enheten för Professionell utveckling, Umeå universitet, Klinisk vetenskap, Umeå, 901 85 Sweden

**Keywords:** Medical education, Empathy, Medical students, Professionalism, Physician-patient relationship, Qualitative study

## Abstract

**Background:**

Empathy has proven to be a fundamental component in the patient-doctor relationship and correlates to several positive outcomes in patient care. Despite this, research suggests that empathy decreases during medical education. To increase the understanding of empathy development during medical education, this study explores medical students’ experiences of learning empathy in the transition from theoretical to practical context.

**Methods:**

Eleven semi-structured interviews with students at a medical school in Sweden. The interviews were transcribed verbatim and analysed using grounded theory.

**Results:**

The analysis resulted in three categories and a core category “Lost in translation”. Early on, students regard empathy as a valued and necessary skill. While students generally encounter high expectations of being empathic, they also met gendered expectations. There is a mismatch between the theoretical and the practical teaching of empathy. The core category refers both to the students feeling lost in their own professional development and empathy becoming lost in the translation from theory to clinical practice.

**Conclusion:**

The results describe clashes between theory and clinical reality and the efforts of the students to develop and maintain empathy in this context. To encourage students to develop empathy it is necessary for both educators and practitioners to acknowledge, and attempt to bridge, the gap between the theoretical and the practical curriculum regarding empathy.

**Supplementary Information:**

The online version contains supplementary material available at 10.1186/s12909-024-06385-z.

## Introduction

The ability to empathize is fundamental if we are to understand the feelings and actions of others and share the affective states of people around us [1]. Empathy has proved to be an essential component in the relationship between patient and doctor [2–5], associated with patient satisfaction and compliance [6–9], as well as positive clinical outcomes [3, 9]. Using empathic communicative skills in an effective way may be the best way to improve patient satisfaction and compliance, and thereby patient health [7].

There is no universally accepted definition of empathy [1]. It has proved to be a complex concept that is hard to define. Mercer et al. define empathy as “the ability to understand the patient’s situation, perspective and feelings, to communicate that understanding and to act on that understanding with the patient in a helpful and therapeutic way” [10]. Laughey et al. suggest a “heart-head model” of clinical empathy, by which they mean that clinical empathy has both an affective (heart) and a cognitive (head) component. The first is the deeper, more embracing kind of empathy compared to the cognitive form which is seen more as “acting out” the empathic role rather than being emotionally moved by the patient [11]. In our study the definition concluded by Mercer et al. [10] has served as our point of departure in how to understand empathy.

Empathy is influenced by several factors such as stress, time constraints [2, 12] and the negative emotional state of a patient [11]. The type and severity of a patient’s medical condition has also been shown to influence empathy – patients with serious diagnoses were easier to empathize with [11, 12].

For medical students, insecurities of different kinds can influence the expression of empathy. Medical students report that feeling insecure, due to lack of experience, when taking the medical history or delivering bad news to a patient might have a negative impact on their empathic ability [12].

Several studies show a decrease in empathy in medical students over the years of medical school and residency [13–16]. It has been suggested that exposure to morbidity and mortality, or the stress that comes with increased responsibility for the patient, could contribute to such a decrease [14]. Whatever the cause, a decrease in empathy may affect and threaten the wellbeing and performance of students [17] as well as the quality of the health care provided [14].

Empathic ability can be improved with training [18, 19] and medical students have identified contact with real patients, as well as course elements preparing students for difficult communication situations, as important for the development of empathy [12].

Since earlier research still shows a decline of empathy as students proceed through medical school it is valuable to further explore how students perceive teaching on empathy, both from a theoretical and clinical perspective to better understand potential gaps in how students translate theoretical empathy into practice. The present study aims to illustrate students lived experiences of empathy in medical education with a specific focus on how interactions within their clinical training effects their ability to empathize. This could be helpful to better adapt and improve both the theoretical and the practical teaching of empathy.

## Methods

### Research design

In this interview study, we deployed an analysis inspired by constructivist grounded theory (GT) as described by Charmaz [20]. We chose constructivist GT as our approach because it is well suited to explore processes, experiences and social phenomena in a context [20] and we consider empathy as a process where the contextual aspects need to be considered.

### Setting and participants

Medical students in their final two semesters (10th and 11th) of medical school at a university in northern Sweden were invited via e-mail to participate in the study. Medical studies in the current setting usually takes a total of five and a half years to complete and are divided between pre-clinical years (semesters one to five) and clinical years (semesters six to eleven). The pre-clinical years are more campus based, primarily theoretical, while the clinical years mainly consists of applied medicine and clinical practice. We chose to recruit from the final two semester as they have experienced empathy in both theoretical and clinical contexts thus presumably rendering a richer material than students at an earlier stage of education.

Therefore, purposive sampling was our first recruitment step as we strived to recruit those how’s experience could serve the aim. After receiving information about the study, the students that volunteered to participate were included in the study, thus the recruitment also applied a self-selection sampling. In total, 11 medical students decided to participate. Six students took part in a focus group interview while five interviews were held individually due to participant preference. Initially, we considered the idea of searching for further participants to perform another focus group but as the data analysis started in parallel with the individual interviews, we realised that the material was rich, and that categories and themes seemed to be recurring. Therefore, the individual interviews served as an in-depth complement to the focus group.

The six participants in the focus group were all women, aged between 24 and 38 years, and were all in the 10th semester of medical school. They had a variety of experiences of working in health care, ranging from working as auxiliary nurses (during spare time or summer breaks) to working as junior doctors (in Sweden, medical students may work as junior doctors after completion of the 9th semester). Of the five individual interviewees, three were women and two men, aged between 24 and 27 years. Four of these participants were in the 10th and one in the 11th semester. They had different experiences of working in medical care, with four of the five having worked as junior doctors.

### Data collection

The interviews were conducted in September and October of 2020 by JS (at the time being a medical student) and JvK (PhD student and a resident in oncology). The interviewers had no prior, close relationship to any of the participants. Written consent was obtained before the interviews. The participants were interviewed using a semi-structured approach, in both individual interviews and focus group. In the focus group JS acted as a moderator while JvK took notes and summarized the discussion in the end to ensure all group members had been included. An interview guide (see Supplementary file 1) was used to facilitate both interviews and focus group discussion. The guide was constructed by JvK, AL (PhD and a specialist in family medicine), and OS (PhD and a registered psychologist). The interview guide was informed by the research team’s previous studies on junior and senior doctors and a general knowledge of the research field. Examples of questions from the interview guide include “What is empathy to you?”, “What do you think about empathy in the medical profession?” and “Are there any educational processes that you perceive has contributed to or affected your empathic ability?”. Additional questions were asked, when necessary, in order to clarify a response or receive more detailed responses from the participants [21]. The interviews lasted 45–90 min and were audio-recorded.

### Data analysis

The interviews were transcribed verbatim, and the analysis was inspired by constructivist grounded theory (GT) as described by Charmaz [20]. The GT process is not linear, and a hallmark of it is concurrent data generation and analysis. Therefore, the first interview to be transcribed was coded and analysed while the remaining interviews were being conducted.

The focus group interview was transcribed first, and the analysis therefore started with reading and coding the written material from that interview. The material was coded openly, line by line, and close to content, as part of the initial coding stage in the GT process [20]. The initial codes were then discussed among JS, JvK, AL, and OS. Then codes were organized into provisional subcategories and categories, as part of the intermediate coding in the GT process. After this, the transcribed materials from the individual interviews were read and coded. The codes from the individual interviews were compared to the initial codes from the focus group interview, and new codes were generated in addition to the previous ones. The new codes generated by the individual interviews were then discussed among all the authors. The agreed codes were then arranged into subcategories and categories. After that, JVK reread the interviews to compare the generated categories with the data to make sure they were grounded in the data. As a part of the advanced coding stage of the GT process, the three categories were compared and related to each other. The categories and how they were related then inspired us to create a core category, encompassing all the subcategories and categories, grounded in the research data from the interviews.

### Reflexive considerations

Group discussion and reflection were essential to the process of defining the research question, recruiting participants, collecting, and analysing data. Through the keeping of field notes during data collection, the interviewers were made aware of their positioning in relation to the participants and how the data was constructed, which enhanced reflexivity [20]. For example, we discussed the differences in how one interviewer, being a student, interpreted codes compared to the perspectives of the supervisors. During analysis JvK made written memos detailing methodological choices and the interpretation of data that helped to make visible pre-existing knowledge and bias. Constant comparison of the different levels of analysis, in combination with regular discussion prompted by the memos, facilitated the joint decision that theoretical saturation had been obtained. Being four researchers with different professional backgrounds as described above, collaborating throughout the research process increased the trustworthiness of the findings.

## Results

When analysing the students’ experiences of empathy in medical education three categories were constructed (see Table 1). In the process of describing, comparing and relating these categories to each other we defined a core category “Lost in Translation” that encompasses and further interprets the students’ experiences described in the categories.

**Table 1:** Overview of, Core category, categories and subcategories

**Core category **	**Categories**	**Subcategories **
Lost in translation	I believe I am, we need it, but it is difficult? - empathy, a valued skill that must be nurtured	The ability to feel with another person; To understand and identify with the emotions of others; Doing empathy; The effects of empathy; Development of empathy; Internal, external, interpersonal, and personal factors; Strategies to maintain empathy.
	I am under pressure – dealing with high expectations	Internal and external expectations; Expectations based on power and gender; Strategies to appear empathic.
	I am dazed and confused – a mismatch between theory and clinical reality	Theoretical teaching of empathy; Practical teaching of empathy; Senior colleagues as role models.

In category 1” I believe I am, we need it, but it is difficult? – empathy, a valued skill that must be nurtured” the participants perceive themselves as empathic individuals and empathy as expected and necessary skill for doctors, but sometimes difficult to achieve. This relates to the expectations and difficulties described in category 2” I am under pressure – dealing with high exceptions”. This category elaborates how the expectations on doctors to be empathic in clinical practice are affected by internal, external and societal norms. Category 3 “I am dazed and confused – a mismatch between theory and clinical reality” further develops how both individual and societal expectations conflicts with the clinical reality where the students perceive empathy as not prioritized.

The categories and how they relate to each other inspired us to form a core category “Lost in translation”, which refers both to the students actually feeling lost (insecure) in their own professional development and empathy seemingly losing its importance in the translation from theory to clinical reality.

### I believe I am, we need it, but it is difficult? – empathy, a valued skill that must be nurtured

The medical students experienced that empathy, while difficult to define, contained elements of identifying and trying to understand the feelings of another person. Active listening, being truly present, and a human touch whenever appropriate, were also believed to be fundamental in empathy. They also observed that the feeling of empathy towards another generated commitment and a willingness to help. Empathy, according to them, is used to establish trust and create a sense of security for the patient. According to the student’s empathy was easily noticed in social interactions involving both verbal and non-verbal communication, stressing that verbal and non-verbal communication must match for empathy to be genuine.

*“When one expresses empathy*,* then there is a meaning behind the words. If you say that you care*,* then I also feel that you care.” (Interview 1)*

The emotional component of empathy varied in their descriptions from empathy not being genuine without an emotional component, to an emotional component not being an absolute requirement for empathy, as seen when comparing the quote above with the one below.

*“Just because you don’t get emotionally engaged doesn’t mean that you cannot show empathy.” (Interview 2)*

The students seem to have conceptualized empathy as a fundamental skill, that while necessary for professional conduct, may also be influenced by various personal, patient or situational factors. The medical students regarded empathy as an asset that helps bridge the power gap between doctor and patient by increasing trust and a sense of security for the patient. They felt that empathy was changeable and influenced by norms and values conveyed by teachers in theory and in clinical practice. Empathy was also described as influenced by changes in students’ personal life as well as one’s own wellbeing and therefore understood as situational. Negative and unpleasant patients, or patients with self-inflicted or seemingly trivial problems, were believed to be harder to empathize with. Patients sharing more personal information was believed to make it easier to relate to the patient, and therefore also easier to empathize with the patient.

*“As a doctor you have high status*,* and we still have a lot of power. We can ask questions of patients who have never met us before and they can share things with us that they have not shared with anyone else. Therefore*,* I think it is very important to show empathy and show an understanding that it might not be easy to share this information and that some things are hard to talk about and hard to experience and not to just sit there like a machine.” (Interview 1)*

*“I think that my empathic ability is dependent on how I feel in my situation, in life. If I feel emotionally stable, then it is easier to empathize and put myself in another person’s situation.” (Focus group interview).*

*“When you have seen how sick people can be*,* then it can be harder to take more trivial medical issues seriously.” (Interview 4)*

The students had different strategies for maintaining their empathic ability. Talking to classmates and family members, being present and seeing and interacting with the patient in the here and now were examples of such strategies. In difficult interactions with patients, it was believed to be important with self-awareness by looking beyond personal prejudices, and previous experiences, and to reflect on one´s own emotions and behaviours as this affects the patient, they are interacting with.

” *Just to talk freely about it. To make oneself aware of situations where you might have wished that somebody had shown more empathy and to learn from this as well. But I would probably say that it is above all this matter of becoming self-aware and think about how you yourself are acting in the encounter with that person. Am I feeling irritated*,* and does it show that I feel irritated*,* because if that is the case then it also isn’t strange if this is in some way mirrored by the person opposite me.” (Interview 1)*

*” I am thinking of a man whom I initially did not care that much about*,* and it sort of felt like he had himself to blame*,* more or less. But then we chatted for a bit and then you get to hear their stories about growing up and things like that and then I can actually feel a great deal of empathy developing. So this*,* yes both being here and seeing the problem right now*,* but also getting to know a person better*,* can also help*,* I believe.” (Interview 2)*

The situation described above indicates that empathic engagement can benefit from personal engagement and increase during a consultation.

### I am under pressure – dealing with high expectations

With regards to empathy, the students reflected that societal expectations conflicted with the culture of medical training. They expressed a belief that the norms of society assumed that they, as medical students, and future doctors, would be empathic. However, when they entered medical school, they were introduced to a biomedical paradigm which seemed to devalue empathy. During their clinical placements they felt that patients expect an empathic encounter, in which they are seen and heard and not just treated medically. Simultaneously, the students instead felt that the patients receive too little empathy, and that the health care provided by their clinical teachers/role models is distanced and bureaucratic. The students expressed a desire to be empathic. This desire, combined with external expectations, makes them feel pressured into expressing empathy. Situations where strong emotions are present further increase these expectations.

*” So*,* when you apply to the medical studies programme*,* they don’t check those abilities at all*,* instead it is really what you know*,* what your grades are. It feels like it is made clear already there that this is what we value*,* it is your knowledge and how clever you are.” (Focus group interview)*

*” You would perhaps feel pressure*,* for instance when you are in psychiatry or something*,* that there is somebody who is perhaps a bit vulnerable mentally*,* that then perhaps you would be a bit more*,* sort of try to show a bit more empathy and treat that person with a bit more empathy.” (Interview 5)*

The students described having experienced unequal gendered expectations. They felt that there is a stronger demand on women than on male students or colleagues to be attentive and empathic. The students believed that men, in general, are given more leeway regarding their behaviour in social situations – that they had more room to be distant and socially awkward.

*“It feels like women are expected to have an interest in other people and to care about other people*,* to take time for others and to listen more than what I think it is for men.” (Interview 3)*

*“As a young woman I think you are regarded as more emotional and that people expect you to be more caregiving*,* as a female doctor or young woman in health care. So*,* the expectations are greater for women to be empathic compared to men and that it is more accepted for a man not to show empathy*,* just because of gender roles and power relations.” (Interview 1)*

The students had different ideas regarding strategies for dealing with situations when they felt that it was expected, but difficult, to empathize. They described that there is a difference between trying to be empathic and true empathy, that empathy is not about playing a part and not something that should be faked. Empathy was supposed to be “true” and genuine. However, there were also descriptions of faking as a strategy to show empathy in situations where it is difficult to empathize. By trying to use the same verbal and non-verbal communication as one would use when empathy is felt, one can simulate empathy.

*“I think that the expectations and the wish to be empathic are strong enough that you will fake empathy instead of honestly saying that you don’t care. You don’t have anything to lose from faking it either. In some way at least*,* you have an understanding that empathy is important.” (Interview 3)*

*” I suppose I try to fake empathy*,* so that you sort of try to use the same words and expressions that you would do otherwise*,* perhaps. To help the person. You feel a bit sneaky*,* of course*,* or a bit fake. This of course does not feel entirely good. But at the same time*,* you are doing it so that the other person might have a better experience of the encounter.” (Interview 5)*

### I am dazed and confused – A mismatch between theory and clinical reality

When the students reflected upon their own empathic ability during their time at medical school, the spectrum ranged from an experienced decline during medical school to empathy being relatively constant. They also experienced a difference between the pre-clinical and clinical semesters of medical school. During the pre-clinical semesters, empathy, whenever addressed, was highlighted through learning activities like active listening. Further, as their medical knowledge was not yet as developed, they focused on being patient-centred and on the patient’s thoughts, concerns, and expectations.

*” When I meet a patient*,* I might not primarily focus on their feelings and instead perhaps more think about the clinical aspects. But when I was new and perhaps my clinical knowledge was not extensive or took up so much room. Perhaps you then notice the person more*,* for better or worse.” (Interview 3)*

*“I think the emotional side of empathy has probably been toned down*,* but perhaps I*,* yes*,* it is as if I think more about empathy or about how I behave*,* previously it was more spontaneous.” (Interview 2)*

*” Well*,* yes*,* I do believe that it did perhaps almost peak at that point*,* during the final pre-clinical semester. I mean when we did theory of consultation and medical psychology and there was an awful lot of focus on it and since then focus has sort of moved to a lot of other things. I do believe that the level of empathy has gone down a bit during the clinical [semesters] or almost [more] the further you get.” (Focus group interview)*.

When the students transitioned from the pre-clinical to the clinical part of their education, they experienced a mismatch between the theoretical teaching of empathy in the pre-clinical semesters and how empathy was expressed in the clinic. The students provided examples of senior colleagues that were bad at expressing empathy or were even non-empathetic. They also noticed an age difference in the display of empathy in the clinic, where younger doctors take more time to sit down with patients, compared to older, more senior colleagues.

*” Sometimes*,* of course*,* you come up against a type of jargon when you are in a clinic*,* where patients are*,* if not completely dehumanized because that sounds so harsh*,* but it is more that it is so common that you don’t quite think about the patient’s background or situation and instead it is just a patient here for this treatment or something.” (Interview 3)*

*” It feels as if the doctor isn’t quite in the same room but is just sitting doing a job of work and forgetting that they are talking to a person. Perhaps it is not as clear as that. But feeling that you are not seen by the doctor*,* this does of course create a massive sense of discomfort and*,* as a doctor*,* not being aware of that surely is a big sign of lack of empathy. I see this as being really very common. Lack of engagement*,* I suppose that is the most benign but also most common sign that people have become non-empathic.” (Interview 4)*

The students noticed that empathy is generally not prioritized in the clinic, that the patient’s feelings are not a primary concern, and that focus lies on medical knowledge. This is described as a conflict in clinical practice – medical students believe that they deliver better healthcare when they establish a connection with the patient and make time to take the patient’s feelings into consideration, but they do not feel that this is valued by their senior colleagues. Medical students want to be empathic, but they feel that in the clinic productivity is more important than taking a moment to sit down and listen to a patient. In addition, empathy was not perceived as measurable in health care. Therefore, taking the time to sit down with a patient did not count at the end of the day, in comparison with the administrative tasks.

*“I think that in order to work efficiently*,* then it is at the expense of how empathic I am towards the patients.” (Focus group interview)*

*“You are so easily influenced by social contexts – you want to fit in with the surrounding context so if your colleagues act in a certain way then you adopt that behaviour without even reflecting on what it actually means.” (Interview 3)*

The students have felt that factors in their working environment, such as stress, time constraints, group dynamics, and the atmosphere between colleagues affect their ability to empathize. The ability to empathize is believed to be influenced by how comfortable the students feel during clinical practice – the more comfortable they felt, the easier it was to empathize with others and vice versa. At the clinic, the way supervisors introduce medical students to patients was said to make a difference in how comfortable the students felt – for example, being introduced as a future colleague as opposed to just a medical student present at the clinic that day. Students also found that if supervisors were empathic towards them, they in turn had more empathy to show towards someone else.

*“When I am a medical student during the clinical semesters it is as if I am not a person. Just someone in white scrubs. Then my emotional ability is not on top. I feel diminished and I might not have the ability to take in someone else’s emotions.” (Focus group interview)*

The students felt that norms and expectations about how to work as a doctor are set by senior colleagues in the clinic, and these therefore become role models for finding one´s way to how to empathize with patients. The way senior colleagues interact with patients can inspire but also serve as examples of how the students do not wish to interact with their own patients. The students truly value the approval of senior colleagues and strive for affirmation from them, although feedback from senior colleagues is said to be rare. Senior colleagues might also add to the stigmatization of certain patient categories, making it harder to empathize with them.

*“I just made a mental note that if you start acting like this doctor then you should actively start thinking about what you are doing and if there is something else that you want to do in life or make a change*,* because this was a catastrophic patient encounter.” (Interview 4)*

*“It becomes so important for me to get my senior colleagues’ affirmation and for them to think that I have done a good job*,* that I would sooner have their approval than do what makes me feel good.” (Focus group interview)*

## Discussion

**Fig. 1:**
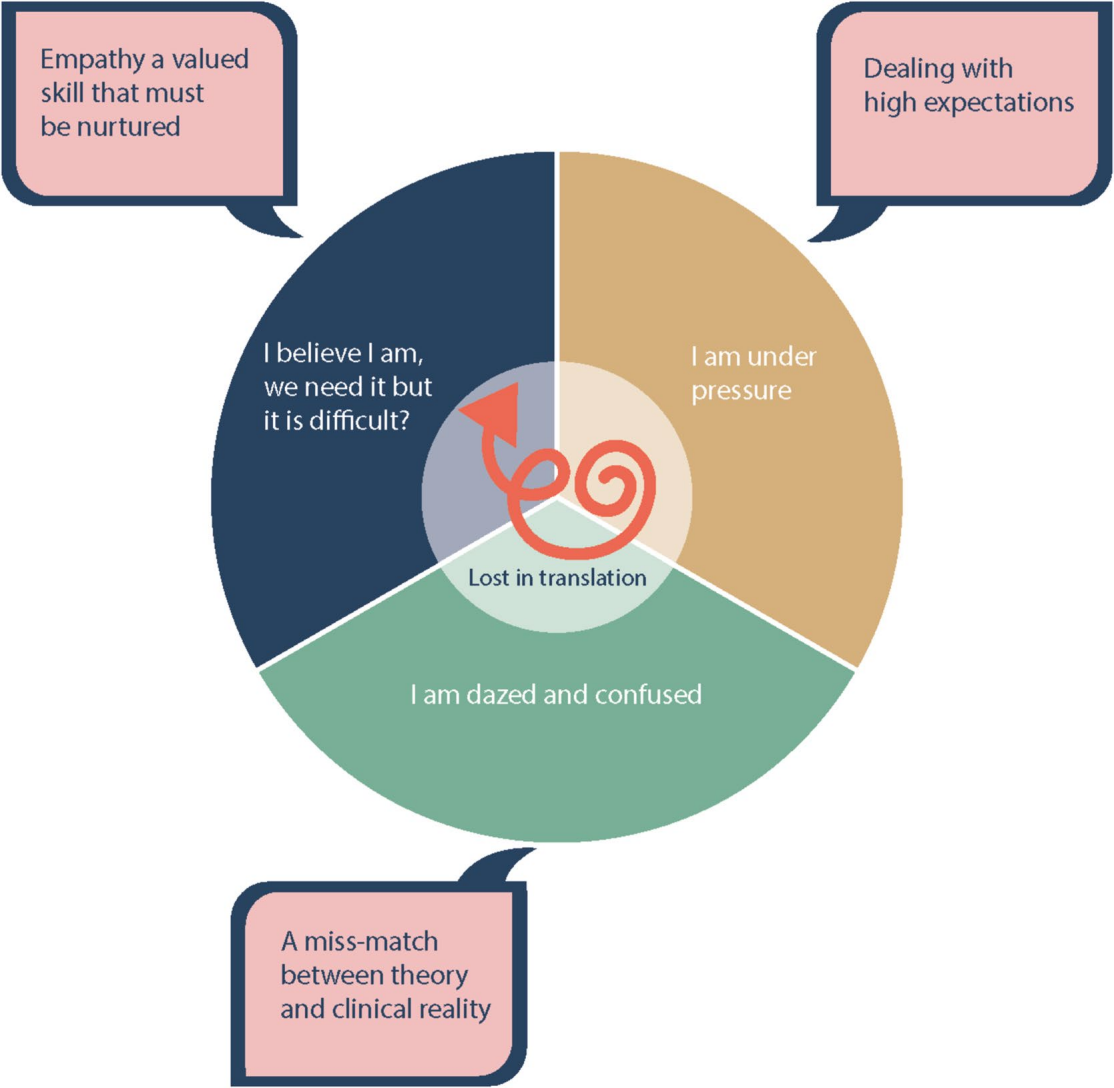
Visual representation of students’ experience of clashes between theory and clinical reality concerning empathy

This study highlights medical students´ lived experience of how empathy is perceived and developed in medical education. The results describe clashes between theory and clinical reality and the students’ efforts to develop and maintain empathy in that context. (Fig. 1).

The medical students regard empathy as something that is difficult to define, which is in line with the findings of earlier studies [10, 22]. They regard empathy as valuable and fundamental in bridging the power gap between doctors and patients. Our results agree with other studies in showing that empathy is influenced by internal factors such as norms and values [2], environmental factors such as stress and time constraints [2, 12], interpersonal factors like lack of common ground, as well as patient factors, where for example patients with a negative attitude are harder to empathize with [12]. While students describe empathy as a skill that requires genuine engagement, there are differences in opinion as to whether the emotional component is actually necessary. Halpern warns that disregarding the emotional component in empathy leads to what she calls detached concern [23]. We would argue that detached concern might decrease doctors’ genuine engagement.

The medical students identified internal as well as external expectations regarding empathy, with the norms of society, the patients, and the students themselves expecting empathy to be highly valued. However, they then enter medical school and find this not to be the case. We interpret this as a clash through which empathy becomes submerged in the biomedical paradigm. This is in line with a recent study, focusing on the role of medical humanities, which described the negative consequences of having a strong biomedical norm in medical education [24]. The students also described different expectations concerning empathy based on gender. A possible consequence of these different expectations is that patients might accept a less empathic encounter with male doctors. Further, male students and doctors might feel less inclined to develop their empathic ability, since both patients and senior colleagues might expect them not to be empathic [25]. We would argue that both these aspects are cause for concern. In our view medical educators need to be aware of the effects of the hidden curriculum as well as gendered expectations and take measures to address them.

Overall, expectations regarding empathy might generate a sense of being pressurized to show empathy. This, in turn, might lead to a preoccupation with one´s own thoughts regarding the display of empathy. Such a preoccupation might make it harder to be present in the here and now, to listen actively and notice non-verbal behaviours that the medical students believed to be fundamental for empathy. Some students mentioned that their strategy for seeming empathic in situations where they did not feel empathic was to feign empathy. They would use empathic statements and the same non-verbal behaviours as one would have used when empathy is felt. This resonates with an earlier study by Laughey et al. about empathy and empathic dissonance [11], where the authors refer to the problem of this “tick-box” empathy by using the novel term empathic dissonance. This term is intended to express the feeling of pressure generated by having to make empathic statements when no “true” empathy is felt.

The medical students believed that lack of knowledge and experience might lead to feelings of insecurity, this is described by Pohontsch et al. [12], which would have a negative influence on empathy. On the other hand, the students in our study also believed that more knowledge and experience would lead to desensitization, making it harder to empathize. This is an interesting and somewhat worrying find, since the medical students still have a long way to go in their medical career. If they are feeling desensitized early in their medical career, this raises the question of what would happen to their empathic ability further on.

When the students entered clinical practice, they experienced a clinical reality where medical knowledge, measurable results, and time management were prioritized over empathic engagement in patient encounters. This mismatch between the predominantly theoretical teaching during the pre-clinical years and the more practical clinical reality concerning empathy is an important finding of the present study. A possible risk of this mismatch is that the students adopt a less empathic approach with their own patients since this is what they see in their senior colleagues. This is problematic since, as has also been found in earlier research [26, 27], medical students regard their senior colleagues as role models for how to empathize with patients. In addition to being role models, senior colleagues can provide valuable feedback to the students regarding their interactions with patients. Feedback and affirmation from senior colleagues were important for the students, but this type of feedback was rare.

The students’ descriptions of the interactions and processes that influence them in their efforts to maintain empathy during medical education aligns with the notion that of empathy as a social construct [28].

The students describe their efforts to develop and maintain empathy as a series of clashes between theory and practice. Previous research has shown a decline in empathy during the transition between pre-clinical and clinical years [13]. In a recent article Laughey et al. suggests that according to the “Heart-head model” there are lots of factors in clinical practice that promote a shift towards a predominantly cognitive (head) empathy instead of affective (heart) empathy, even if affective empathy is seen as a more genuine form of empathy [11]. Our study aligns with the results above regarding that empathy is influenced by several obstacles in the clinical context. However, the students in our study express a global devaluation of empathy in clinical practice rather than a shift from affective to cognitive empathy.

As the students move from the pre-clinical years to the clinical years, this study highlights the difficulty in putting theoretical knowledge into practice. So, rather than being lost in transition due to callousness, our study suggests that medical students’ empathy becomes lost in the translation from theory to clinical reality. This process makes the students dazed and confused in their professional development. Therefore, we argue that the students, as well as empathy, becomes lost in translation.

### Strengths and limitations

The interviewees were all students at the same university which might limit the applicability of the results as the teaching of empathy differs between universities. In the present study, due to self-selection sampling, there is also a possible bias in form of a loss of the perspective of students uninterested in the topic of empathy or who might consider empathic ability to be unimportant. However, we did find a variation in for example how important the affective part of empathy is, suggesting a reasonably diverse sample. The use of two different interview settings in the present study, both a focus group and individual interviews, provided a more in-depth material [29]. We also consider that the methodological rigour, including discussions and reflexivity, have increased the trustworthiness of our results.

## Conclusions

At an early stage, medical students regard empathy as a valued and necessary skill. This ideal causes pressure, and the sense of it being a requirement sometimes leads to students faking it. While students generally experience high expectations of being empathic, there are also gendered variations, with male students being given more leeway. There is a mismatch between the attitude towards empathy during pre-clinical years and the attitude encountered in the clinic. Students feel that empathy is devalued in the clinic, in favour of productivity. Medical students might also adopt a less empathic way of interacting with their patients after observing senior colleagues who do not show empathy towards patients. This poses a threat to the quality of health care.

This study highlights the importance of creating an environment where students are encouraged to develop their empathic ability, for example through reflective learning activities. Medical education also needs to include learning activities that address gendered expectations and differences in empathy.

The findings of this study also indicate a need for senior colleagues to reflect upon their own position as role models able to involve medical students in reflections regarding interactions with patients. Further, health care organizations need to enable time for empathic interactions and possibilities for reflection to foster empathy.

In sum, educators, practitioners as well as policy makers in health care need to acknowledge and attempt to bridge the gap between the theoretical and the practical curriculum that regards empathy.

Future research on empathy should investigate how to prepare medical students for their future work in the clinic and how to help them maintain their empathic ability in daily clinical life. For example, the effects of interventions targeting empathy and gendered expectations on empathy.

Further, there might be a need for investigating the consequences of faking empathy, if there are any dangers or if any of the positions of the “heart-head model” is preferable. Also, the possible connections between faked empathy and for example burnout needs to be understood.

## Supplementary Information

Supplementary Material 1.

## Data Availability

The interview transcripts are not publicly available due to reasons of confidentiality. Data are however available from the corresponding author on reasonable request.
