# Supplementary material for: Lost in translation: a qualitative study of medical students’ experiences of theoretical and practical teaching of empathy
Source: BMC Med Educ. 2024 Dec 4;24:1416. doi: 10.1186/s12909-024-06385-z (PMC11616379; doi:10.1186/s12909-024-06385-z)
Supplement: Supplementary file 1 — Supplementary Material 1. [file 12909_2024_6385_MOESM1_ESM.docx]

**Supplementary file 1: Interview guide: Empathy, medical students’ perspective.**

**Understanding of empathy as a concept, general ideas**

What is empathy to you?

How do you notice if someone is empathic?

Can you describe situations where empathy takes place?

**Empathy in education and medical profession**

What do you think about empathy in the medical profession, does empathy matter in the doctor-patient relationship?

How do you feel about your own empathic ability - Are there any educational processes that you perceive has contributed to or affected your empathic ability?

Have you experienced that medical students are expected to always be empathic? Have you experienced that the expectations differ depending on for example gender and age?

Have you experienced situations where you are pressured to show empathy without really feeling any empathy? How does that make you feel and how do you handle that?

What do you expect from your future work life, are there any obstacles or possibilities for being empathic?

Final question, after been given a summary: Is there anything you would like to add?
